# Role of CT scan-based and clinical evaluation in the preoperative prediction of optimal cytoreduction in advanced ovarian cancer: a prospective trial

**DOI:** 10.1038/sj.bjc.6605292

**Published:** 2009-09-08

**Authors:** G Ferrandina, G Sallustio, A Fagotti, G Vizzielli, A Paglia, E Cucci, A Margariti, L Aquilani, G Garganese, G Scambia

**Affiliations:** 1 Gynecologic Oncology Unit, Division of Gynecologic Oncology, Department of Oncology, Catholic University of the Sacred Heart, Campobasso, Italy; 2 Department of Imaging, Catholic University of the Sacred Heart, Campobasso, Italy; 3 Division of Gynecologic Oncology, Department of Obstetrics/Gynecology, Catholic University of the Sacred Heart, Rome, Italy

**Keywords:** prospective trial, ovarian cancer, cytoreduction, predictive models, CT scan

## Abstract

**Background:**

In advanced ovarian cancer, maximal efforts have to be attemptedto achieve optimal cytoreduction, as this represents the keystone in the therapeutic management. This large, prospective study aims at investigating the role of computed tomography (CT) scan in predicting the feasibility of optimal cytoreduction in ovarian cancer.

**Methods:**

A total of 195 consecutive patients with clinical/radiographic suspicion of advanced ovarian/peritoneal cancer were enrolled at the Gynecologic Oncology Unit, Catholic University of Rome and Campobasso, Italy. Preoperative CT scans were performed with a high-speed scanner (CT Hi Speed Nx/i Pro; 2-slice; GE Medical System). All patients underwent standard laparotomy, and maximal surgical effort was attempted. The following CT parameters were used: peritoneal thickening, peritoneal implants >2 cm, bowel mesentery involvement, omental cake, pelvic sidewall involvement and/or hydroureter, suprarenal aortic lymph nodes >1 cm, infrarenal aortic lymph nodes >2 cm, superficial liver metastases >2 cm and/or intraparenchimal liver metastases any size, large volume ascites (>500 ml). Clinical data included were age, Ca125 serum levels, and ECOG-PS. Radiographic and clinical features exhibiting a specificity >75%, a positive and negative predictive value >50%, an accuracy >60% in predicting surgical outcome were assigned a point value of 2. With this scoring system, a predictive index (PI) was calculated for each patient.

**Results:**

The PI scores ranged from 0 to 6, and from 0 to 8, in Model 1 (including only radiographic parameters) and in Model 2 (including radiographic and clinical data). The AUC was 0.78+0.035 in Model 1, and 0.81+0.031 in Model 2. Therefore, the addition of ECOG-PS data led to the improvement of the diagnostic performances (*z*=2.41, *P*-value <0.05).

**Conclusions:**

Computed scan still represents a valid tool to predict ovarian cancer optimal cytoreduction; the predictive ability of a CT scan-based model is improved by integrating ECOG-PS data.

Even in the absence of randomized trials supporting the role of cytoreductive surgery in the treatment of advanced ovarian cancer, there is a general consensus regarding the need to pursue the achievement of the minimal amount, or even the absence of visible residual tumour at primary surgery, given its direct relation with prolonged survival time (Hunter *et al*, 1992; Bristow *et al*, 2002; Eisenkop *et al*, 2006).

Although the term ‘optimal’ has been applied over time to cytoreductive surgery achieving a maximal diameter of residual tumour from 0 to even 3 cm (Nickles Fader and Rose, 2007), the GOG currently defines as ‘optimal’ a residual disease ⩽1 cm; indeed, the evolution of this definition to include patients undergoing cytoreduction to no apparent disease is reasonably going to rapidly occur (Bristow *et al*, 2002). Therefore, while awaiting for the mature results of the EORTC 55971 phase III randomized trial comparing upfront debulking *vs* secondary cytoreduction after neo-adjuvant chemotherapy in stage IIIC/IV ovarian cancer (Vergote *et al*, 2008), maximal efforts have to be attempted to leave no residual tumour at primary surgery, as this commitment represents the keystone in the management of advanced disease.

In this context, much attention has been focused on laboratory assay, clinical and radiographic parameters, or, more recently, laparoscopically assessed scores (Fagotti *et al*, 2006, 2008; Brun *et al*, 2008) able to preoperatively define each patient's chance to undergo optimal cytoreduction. Although the accuracy of preoperative serum Ca125 levels ranges between 50 and 78%, and conflicting data about the predictive ability of Ca125 levels have been reported (Chi *et al*, 2000, 2009; Cooper *et al*, 2002; Saygili *et al*, 2002; Memarzadeh *et al*, 2003), computed tomography (CT) scan assessed parameters might conceivably offer better predictive performances, as they define not only the extension of disease, but also more important, the involvement of specific intra-abdominal sites generally recognised to heavily preclude the feasibility of optimal debulking, such as portal triad disease, agglutinated bowel/mesentery, bulky diaphragmatic disease, or suprarenal aortic lymph nodes (Eisenkop and Spirtos, 2001). In particular, Bristow *et al* (2000) developed a CT scan-based model achieving an overall accuracy of 93% in predicting successful cytoreduction. However, the recent demonstration that CT predictors are not reliably reproducible in series different from the one(s) upon which the model was originated has questioned the true role of CT scan in predicting surgical outcome outside each own institution (Axtell *et al*, 2007). Additional sources of concern about the usefulness of CT imaging predictors are represented by the small size of previously published cohorts, often including early-stage cases, their retrospective nature, and heterogeneity of imaging procedures across institutions, as well as combinations of different CT predictors (Nelson *et al*, 1993; Forstner *et al*, 1995; Meyer *et al*, 1995; Bristow *et al*, 2000; Byrom *et al*, 2002; Dowdy *et al*, 2004; Qayyum *et al*, 2005; Axtell *et al*, 2007).

Moreover, discrepancies across the studies and reliability of the results are also conceivably related to the time frame, and duration of accrual as variations and/or improvements in the imaging techniques, equipment, and performances have occurred over time, and also diverge across different imaging centres.

The aim of this study was to investigate the overall performance of CT in predicting the feasibility of primary optimal cytoreduction in advanced ovarian cancer patients in a large, prospective trial. The performances of different predictive models, including also clinically assessed parameters, have been considered.

## Materials and methods

Between January 2005 and October 2008, 195 consecutive patients with clinical and radiographic suspicion of advanced (Stage III–IV) ovarian/peritoneal cancer were consecutively enrolled at the Gynecologic Oncology Unit, Catholic University of Rome and Campobasso, Italy.

Routinary staging work up included complete physical and gynaecological examination, Ca125 serum level assessment, chest X-rays, and abdomino-pelvic CT scan. ECOG performance status (ECOG-PS) was also recorded. To select advanced stage cases, clinical and radiological parameters were used as the presence of at least two of the following criteria: ascites (>500 ml), CT evidence of metastatic disease, and elevated Ca125 levels (>500 IU ml^−1^).

Exclusion criteria were represented by ECOG-PS >2, large volume extra-abdominal disease.

At time of study conception and design (2004), the approval of the Institutional Review Board was not required because the study did not include diagnostic or therapeutic procedures different from the standard ones (preoperative staging and cytoreductive surgery).

### Imaging technique

Preoperative CT scans were performed with a high-speed scanner (CT Hi Speed Nx/i Pro; 2-slice; GE Medical System, Milwaukee, WI, USA). Computed tomography examinations were obtained after the oral administration of 1000 ml of diluted iodinated water soluble contrast medium (approximately 2% Gastrografin solution; 20 ml Gastrografin/1000 ml water). All CT scans were acquired at baseline and 70 s after i.v. administration of 120–130 ml high-concentration non-ionic iodinated contrast medium (350–370 mgI ml^−1^). The usual flow rate was 3 ml s^−1^.

Images were obtained in a craniocaudal direction, from diaphragm to the ischial tuberosities, with 5 mm thickness and 15 mm s^−1^ table speed. The hard copy images were reviewed by two radiologists (GS, EC) with a special interest in gynaecologic oncology imaging, who were unaware of the clinical characteristics of the patients. In case of disagreement a re-joint evaluation of the scans was performed until a consensus was reached.

### Surgical procedures

All patients underwent standard longitudinal laparotomy, and intensive surgical staging was attempted according to the standard guidelines. Maximal surgical effort (achievement of <1 cm residual disease) has been attempted in all patients, and when possible, included surgical removal of tumour masses, along with total abdominal hysterectomy, bilateral salpingo-oophorectomy, radical omentectomy, appendectomy, multiple biopsies, and additional surgery (intestinal resections (20%), diaphragm stripping (20%), abdomino-pelvic peritoneal stripping (35%), liver and pancreatic resection (9%), splenectomy (15%), if required. Radical pelvic and para-aortic lymphadenectomy was performed in all patients undergoing primary cytoreduction leaving a residual tumour ⩽1 cm. In case of impossibility to proceed to primary optimal cytoreduction, as assessed at primary laparotomic effort, patients were triaged to neoadjuvant chemotherapy (Fanfani *et al*, 2003; Vergote *et al*, 2005).

### Data analysis

The computed tomography parameters used in the data analysis were as follows: peritoneal thickening (diffuse, linear, >1 cm thickening) or peritoneal implants >2 cm, bowel mesentery involvement, omental extension (spleen, stomach, lesser sac), pelvic sidewall involvement and/or hydroureter, suprarenal aortic lymph nodes >1 cm, infrarenal-aortic lymph nodes >2 cm, superficial liver metastases >2 cm and/or intraparenchimal liver metastases any size, large volume ascites (>500 ml).

Clinical data used in the analysis were age, Ca125 serum levels, and ECOG-PS. For the purpose of the study, analysis of the data was performed by two different approaches: in Approach A, the sensitivity, specificity, negative predictive value (NPV), positive predictive value (PPV), and accuracy of each radiographic parameters, as well as clinical features in predicting surgical outcome were calculated. Sensitivity was defined as the number of correctly defined suboptimally debulked cases (true positives) divided by the total number of suboptimally cytoreduced patients. Specificity was defined as the number of correctly defined optimally debulked cases (true negatives) divided by the total number of optimally cytoreduced patients.

NPV corresponded to the number of true negatives divided by the total number of negative results for each parameter, and PPV corresponded to the number of true positives divided by the total number of positive results for each parameter. Accuracy was calculated as the sum of true positives and true negatives divided by the total number of patients in the study.

Inclusion of a specific radiographic parameter in the final model required a specificity ⩾75%, a PPV ⩾50%, and a NPV⩾50%: the radiographic feature satisfying these three criteria was assigned a point value of 1 (Bristow *et al*, 2000). An additional point was assigned to those parameters that, besides the above criteria, also showed an overall accuracy ⩾60% in predicting surgical outcome. With this scoring system, a predictive index (PI) was calculated for each patient.

In Approach B, the *χ*^2^-test or Fisher's exact test for proportion were performed for each radiographic and clinical parameter, and all features showing a statistically significant association (*P*-value <0.05) with surgical outcome were analysed by means of logistic regression (Cox, 1970) using a stepwise routine. Features shown to maintain the association with surgical outcome in multivariate analysis were used to generate a predictive model. In particular, a PI score was assigned to each patient according to the absence or presence of any of the variables identified. The predictive performances of the PI scores in approaches A and B were tabulated in different categories, and receiver operating characteristic (ROC) curves were obtained to analyse the ability of different PI models in predicting surgical outcome. The statistical significance of differences between ROC estimates was performed applying the method by Hanley and McNeil (1982).

Finally, the pre-test probability, likelihood ratios, and post-test probability of models deriving from Approaches A and B were calculated to assess their efficacy in predicting surgical outcome (Deeks and Altman, 2004).

## Results

At the end of enrolment, the final study included 195 patients consecutively seen at the Division of Gynecologic Oncology of the Catholic University of Rome and Campobasso; patients’ characteristics at initial diagnosis as well as their surgical outcome, and final pathology are summarised in Table 1. Median age was 59 years (range: 31–85), with 69 (35.4%) of cases aged ⩾65 years; approximately 25% of patients had ECOG-PS=2. Overall, the rate of cytoreduction to absent or ⩽1 cm residual disease were 27.2 and 16.9%, respectively, for an overall proportion of optimal cytoreduction of 44.1%. One hundred seventy-four (89.2%) were diagnosed as having primary ovarian carcinoma, whereas 19 cases (9.7%) were metastatic tumours from other primary tumours, thus emphasising the need to proceed always to the histological assessment of pathology. Because of the prospective nature of the study, results refer to the whole study population. One hundred forty-five patients (74.4%) had CA125 serum levels >500 IU ml^−1^.

Neither was there any difference in the percentage of optimal cytoreduction over time of enrolment, nor across the six operating teams (data not shown).

### Imaging findings

The features assessed on CT scan were prospectively recorded in the data form presented in Table 2. In the same Table the diagnostic performances of each radiographic parameter compared with laparotomic findings are summarised: in terms of specificity, the best performance was documented for infrarenal aortic lymph nodes and omental extension, whereas very low specificity rates were documented for peritoneal thickening and ascites. Overall, the accuracy rate ranges between 40.2 (pelvic involvement) and 81.9% (involvement of infrarenal aortic lymph nodes).

### Approach A

**Figure 1:**
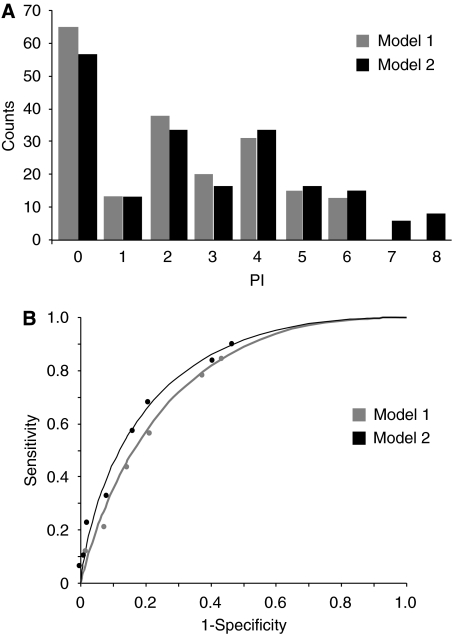
Distribution of predictive index values (**A**) and ROC curves (**B**) in Model 1 and Model 2.

According to the Bristow criteria, two radiographic parameters (omental extension, liver involvement) fulfilled the criteria required for being assigned a point value=1, while the involvement of diaphragm or bowel mesentery, and, among clinical parameters, ECOG-PS obtained a point value of 2 (Table 3). On the basis of the absence or presence of the above cited parameters a PI score was calculated for each patient; the frequency distribution of the predictive score in the overall series are presented in Figure 1. In particular, the PI scores range from 0 to 6 (median=2) and from 0 to 8 (median=2) in Model 1 (not including ECOG-PS data) and in Model 2 (including ECOG-PS data), respectively. The calculation of sensitivity, and specificity was carried out for each PI score ⩾1 to the upper limit in each model, and the ROC curve analysis was performed. The AUC was 0.78±0.035 in Model 1, and 0.81±0.031 in Model 2.

The addition of ECOG-PS data led to the improvement of the diagnostic performances, as the difference between the AUCs of Model 1 and Model 2 was statistically significant (*z*=2.41, *P*-value <0.05).

### Approach B

Univariate and multivariate analysis were carried out to analyse radiographic and clinical features for their association with surgical outcome (Table 4). All radiographic parameters but one (pelvic involvement) were shown to be predictive of residual disease in univariate analysis, and were therefore included in the multivariate analysis.

**Figure 2:**
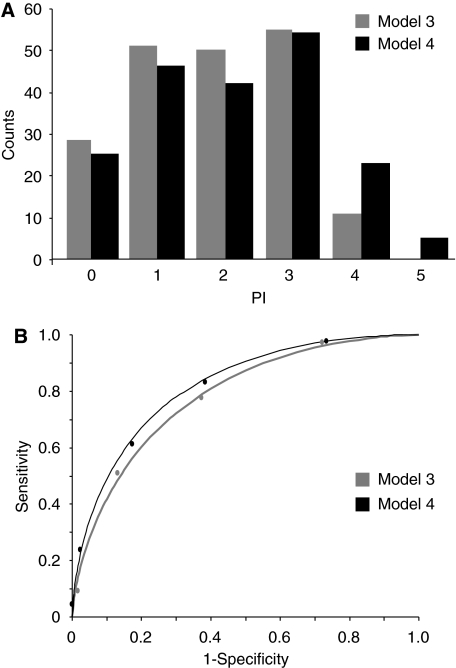
Distribution of predictive index values (**A**) and ROC curves (**B**) in Model 3 and Model 4.

Among clinical parameters, only ECOG-PS was associated with the extent of primary cytoreduction. In multivariate analysis, only involvement of peritoneum, bowel mesentery, suprarenal aortic lymph nodes, and diaphragm, as well as ECOG-PS maintained their independent association with surgical outcome, and were assigned 1 point value. The PI score therefore ranged from 0 (absence of all selected radiographic features) to 4 (presence of all selected radiographic features) in Model 3, and from 0 to 5 in Model 4, which included selected radiographic variables plus ECOG-PS. Figure 2 shows the distribution of PI scores in Models 3 and 4. The calculation of sensitivity and specificity was carried out for each PI score ⩾1 to the upper limit in each model, and the ROC curve analysis was performed. The AUC was 0.78±0.034 in Model 3, and 0.82±0.031 in Model 4. In this case, also the addition of ECOG-PS data produced a more favourable AUC for Model 4 (*z*=3.41, *P*-value <0.05).

The pre-test probability, likelihood ratio, and the post-test probability were calculated for Models 2 and 4 at different cutoff values (Table 5). The pre-test probability was 55.8% (109 patients with residual tumour >1 cm at primary cytoreductive effort).

There was an increasing improvement of the post-test probability paralleling the increase in the cutoff values for both Models 2 and 4: indeed in Model 2, 27 cases had a PI score >5, and 25 of them had suboptimal cytoreduction; the positive likelihood ratio was 9.86, and the post-test probability was 92.6% with an improvement of 36.8% compared with the pre-test probability.

Similarly, in Model 4, 28 cases had a PI score >3, and 26 of them had suboptimal cytoreduction; the positive likelihood ratio was 10.25, and the post-test probability was 92.8% with an improvement of 37.0% compared with the pre-test probability.

To take advantage of a ‘easy handling’ PI score, practically, the rate of inappropriate unexploration, which therefore should tend to 0, can be easily calculated as the inverse of PPV, whereas the proportion of unnecessary exploration corresponds to the inverse of NPV. In our series, only Models 2 and 4 (both including radiographic features and ECOG-PS data) provided PPVs=100% at PI score cutoff values of 7 and 4, respectively (Tables 6 and 7).

## Discussion

This is the first study reporting the results from a very large, prospective trial investigating the role of CT scan-based evaluation in the preoperative prediction of optimal cytoreduction in advanced ovarian cancer. We developed predictive models from two different approaches (on the basis of either on diagnostic performance of each CT-derived parameter, or on results from multivariate analysis), which also took significant clinical variables into account: in both approaches ECOG-PS resulted the only clinical variable fulfilling the criteria required for inclusion in the predictive models, whereas preoperative Ca125 serum levels and age showed a low degree of accuracy in predicting surgical outcome. Indeed, the predictive models, including ECOG-PS data, were more accurate than those derived from CT alone; these findings confirm previously reported results (Bristow *et al*, 2000; Chi *et al*, 2000; Cooper *et al*, 2002; Saygili *et al*, 2002; Memarzadeh *et al*, 2003), and definitively recognise the extent of the impact played by ECOG-PS in the preoperative prediction of ovarian cancer primary resectability (Aletti *et al*, 2007). The models based on diagnostic performance and on results from multivariate analysis showed the same accuracy in predicting the chance of optimal cytoreduction, although they included slightly different CT-based features; in particular, involvement of bowel mesentery, omentum, liver, and diaphragm were shown to fulfil all the required criteria (Bristow *et al*, 2000) in Approach A, whereas in multivariate analysis the involvement of peritoneum and suprarenal aortic lymph nodes, besides bowel/mesentery and diaphragm disease, were independently associated with suboptimal cytoreduction. The divergence between the two approaches remains difficult to explain, although the strict and, to a certain extent, unpredictable associations among the variables might more likely have an impact on multivariate analysis. In any case, our findings support the relevance of the assessment of the status of bowel mesentery and diaphragm involvement, recognised among the most important features determining the feasibility of ovarian cancer cytoreduction (Bristow *et al*, 2000; Axtell *et al*, 2007). Although the results obtained using the two approaches were similar, we think that the approach based on diagnostic performances of CT parameters plus ECOG-PS is more easily understandable and manageable, and is therefore recommended for future studies and/or use.

A direct comparison of our results with those reported in the literature is rather difficult; indeed, the vast majority of previously published studies investigated relatively small sample series and, given their retrospective design, were likely characterized by a selection bias (Table 8). Moreover, the number of CT-assessed parameters used in the prediction of surgical outcome showed a wide range of variability across different studies (Nelson *et al*, 1993; Forstner *et al*, 1995; Meyer *et al*, 1995; Bristow *et al*, 2000; Byrom *et al*, 2002; Dowdy *et al*, 2004; Qayyum *et al*, 2005; Axtell *et al*, 2007) and, more important, the use of a CT-based cutoff score was investigated only by three Institutions (Nelson *et al*, 1993; Forstner *et al*, 1995; Eisenkop and Spirtos, 2001). Nonetheless, there is a general consensus that CT scan represents a valid tool to address the issue of preoperative prediction of ovarian cancer resectability at primary surgery.

Some issues, however, have to be discussed: first, although it can be argued that the percentage of optimal cytoreduction in our series was not so close to the upper limit of the range reported in the literature (Nickles Fader and Rose, 2007), it has to be taken into account that our patients were selected on the basis of clinical and radiographic features of very advanced disease, thus resulting in 91.3% FIGO stage IIIC/IV cases, whereas other series also included FIGO stage I/II patients in a range between 19 and 45% (Nelson *et al*, 1993; Forstner *et al*, 1995; Meyer *et al*, 1995; Byrom *et al*, 2002; Qayyum *et al*, 2005). We recognise that the predictive performance of any test is expected to loose a part of its potential advantages with an increasing rate of optimal cytoreduction. Although this remains to be experimentally tested, on the other hand it cannot be excluded that changes in the percentage of optimal cytoreduction across different centres could more likely result in the modification of the threshold level of the PI cutoff value, rather than in questioning the overall PIV-based approach. Moreover, it has to be acknowledged that very high percentages of optimal cytoreduction are hardly achievable outside a few, very committed Institutions; in this context, the availability of a tool suitable to adapt to the range of the most commonly achievable rates of optimal cytoreduction is clinically relevant (Wakabayashi *et al*, 2008).

We developed predictive models able to produce different PI values, thus providing the chance to choose the most adequate cutoff on the basis of patients’ and disease characteristics (performance status, need to perform very extensive surgery), and surgeon's commitment: obviously, the predictive performance of any model varies with the chosen cutoff value of PI; for instance, if we had used a PI value of 2 (see Table 6, Model 2), we would have obtained a rate of unnecessary exploration of 33.3%, in face of a rate of inappropriate unexploration of 19.4%, which means that almost one-fifth of our patients would have been deprived of the potential survival benefits achievable with optimal cytoreductive surgery.In this context, the need to use a PI with the highest degree of accuracy in minimising the rate of cases erroneously judged to have an unresectable disease is of utmost important, and is even more relevant than running the risk of unnecessarily explore patients who rather present unresectable disease at laparotomy. Therefore, as a practical rule, the calculation of the rate of inappropriate unexploration can be carried out from our models, as the inverse of PPV and therefore will be 0% at the cutoff values of 7 and 4, in Models 2 and 4, respectively. Finally, more sophisticated imaging approaches such as Positron emission tomography/computed tomography (PET/CT) (Risum *et al*, 2008), as well as laparoscopic approaches (LPS) scores (Fagotti *et al*, 2006, 2008; Brun *et al*, 2008) have been recently investigated in terms of prediction of surgical outcome in advanced ovarian cancer, whereas the results of PET/CT seem currently too preliminary to draw any definitive conclusion, data from pilot and prospective studies proposed open LPS as a reliable and flexible predictive tool scores (Fagotti *et al*, 2006, 2008; Brun *et al*, 2008). Although the accuracy of LPS in the assessment of specific sites of disease involvement is expectedly higher compared with CT scan (Fagotti *et al*, 2006, 2008; Brun *et al*, 2008), the clinical impact of whether triaging or not advanced ovarian cancer patients to laparotomy on the basis of LPS findings urgently requires to be investigated in controlled clinical trials.

In conclusion, we showed that CT scan still represents a valid tool into address the issue of preoperative prediction of ovarian cancer resectability at primary surgery, and that its predictive performances might be improved by the inclusion of ECOG-PS data. As already acknowledged (Bristow *et al*, 2000; Cooper *et al*, 2002), a multi-institutional prospective trial hopefully integrating preoperative clinical and radiographic variables is required to test whether the predictive models maintain their accuracy when applied to different patient cohorts. Indeed, a very recently published study by Gemer *et al* (2009) has underscored the difficulty to devise generally applicable models able to reliably predict surgical outcome in advanced ovarian cancer patients, across different Institutions. This issue can become clinically more relevant in the light of the upcoming mature results from EORTC 55971 trial.

## Figures and Tables

**Table 1:** Characteristics of the patients enrolled

**Characteristics**	**No. of patients (%)**
All cases	195
	
*Age (years)*	
⩽65	126 (64.6)
>65	69 (35.4)
	
*ECOG-PS*	
0–1	146 (74.9)
2	49 (25.1)
	
*Residual tumour*	
Absent	53 (27.2)
⩽1 cm	33 (16.9)
>1 cm	109 (55.9)
	
*Final pathology*	
Ovarian cancer	174 (89.2)
Synchronous tumour (endometrial, breast)	2 (1.0)
Other primary^a^	19 (9.7)
	
*FIGO stage* b	
IIIB	17 (8.7)
IIIC	142 (72.8)
IV	36 (18.4)
	
*Ca125 levels (IU ml* ^*−1*^ *)*	
⩽500	50 (25.6)
>500	145 (74.4)

a Sarcoma (*n*=5), stomach (*n*=4), colorectal (*n*=4), endometrial (*n*=3), breast (*n*=2), lymphoma (*n*=1).

b Only ovarian cancer.

**Table 2:** Accuracy, negative, and positive predictive value of computed tomography scan assessed parameters *vs* laparotomic findings

	**Specificity**	**NPV**	**PPV**	**Accuracy**
Peritoneal thickening, peritoneal implants >2 cm	47.5	48.7	86.2	78.5
Bowel mesentery involvement	69.6	83.3	61.1	72.6
Omental extension (spleen, stomach, lesser sac)	87.0	87.6	42.8	79.2
Pelvic sidewall involvement and/or hydroureter	81.0	30.9	73.8	40.2
Suprarenal aortic lymphnodes >1 cm	85.9	75.3	50.0	70.3
Infrarenal-aortic lymphnodes >2 cm	91.8	86.6	50.0	81.9
Superficial liver metastases >2 cm and/or intraparenchimal liver metastases any size	80.9	85.6	46.1	74.9
Large volume ascites (>500 ml)	55.7	84.5	70.9	75.0
Diaphragmatic disease (widespread infiltrating carcinomatosis, or confluent nodules)	90.7	46.2	93.9	67.0

**Table 3:** Prediction of optimal cytoreduction: computed tomography-based and clinically assessed parameters assigned a point value according to Bristow criteria (Approach A)

	**Specificity**	**NPV**	**PPV**	**Accuracy**	**Point**
*Radiographic*					
Peritoneal thickening, peritoneal implants >2 cm	34.5	**74.3**	**63.8**	**65.9**	0
Bowel mesentery involvement	**75.4**	**61.8**	**73.6**	**67.5**	**2**
Omental extension (spleen, stomach, lesser sac)	**92.9**	**50.4**	**82.8**	56.2	**1**
Pelvic sidewall involvement and/or hydroureter	**80.2**	45.6	**59.5**	48.7	0
Suprarenal aortic lymph nodes >1 cm	**86.0**	47.7	**70.0**	**52.3**	0
Infrarenal aortic lymph nodes >2 cm	**94.2**	48.5	**82.1**	**53.3**	0
Superficial liver metastases >2 cm and/or intraparenchimal liver metastases any size	**83.5**	**51.1**	**73.1**	57.1	**1**
Large volume ascites (>500 ml)	40.7	**60.3**	**62.2**	**61.6**	0
Diaphragmatic disease (widespread infiltrating carcinomasis, or confluent nodules)	**77.6**	**61.1**	**76.8**	**67.8**	**2**
					
*Clinical*					
Age, years (⩽65 *vs* >65)	73.2	48.4	**64.6**	53.1	0
Ca125 levels, IU ml^−1^ (⩽500 *vs* >500)	25.8	44.8	**55.9**	53.3	0
ECOG-PS (0–1 *vs* 2)	**91.9**	**54.1**	**85.7**	**62.0**	**2**

Statistically significant values have been indicated as bold values.

**Table 4:** Prediction of optimal cytoreduction: univariate and multivariate analysis by logistic regression of CT-based and clinically assessed parameters to use for modeling (Approach B)

	**Univariate**		**Multivariate**		
	***χ*** ^**2**^	***P*-value**	***χ*** ^**2**^	***P*-value**	**Point^a^**
*Radiographic*					
Peritoneal thickening, peritoneal implants >2 cm	16.34	**0.0001**	**3.45**	**0.063**	**1**
Bowel mesentery involvement	17.50	**0.0001**	**5.35**	**0.0207**	**1**
Omental extension (spleen, stomach, lesser sac)	11.13	**0.0008**	**2.52**	0.11	0
Pelvic sidewall involvement and/or hydroureter	2.28	0.13	**—**	**—**	0
Suprarenal aortic lymph nodes >1 cm	4.21	**0.040**	4.36	**0.036**	**1**
Infrarenal aortic lymph nodes >2 cm	8.15	**0.0043**	**—**	**—**	0
Superficial liver metastases >2 cm and/or intraparenchimal liver metastases any size	8.58	**0.0034**	**—**	**—**	0
Large volume ascites (>500 ml)	8.15	**0.0043**	**—**	**—**	0
Diaphragmatic disease (widespread infiltrating carcinomasis, or confluent nodules)	25.35	**0.0001**	**7.13**	**0.0076**	**1**
					
*Clinical*					
Age, years (⩽65 *vs* >65)	2.51	0.08	**—**	**—**	0
Ca125 levels (⩽500 *vs* >500 IU ml^−1^)	2.98	0.11	**—**	**—**	0
ECOG-PS (0,1 *vs* 2)	19.71	**0.0001**	**14.22**	**0.0002**	**1**

a Only variable achieving a *P*–value <0.10 were assigned a point value. Statistically significant values have been indicated as bold values.

**Table 5:** Pre-test probability, likelihood ratio, and post-test probability for different predictive models of primary optimal cytoreduction in ovarian cancer

**Test**	**Cutoff**	**Pre-test probability**	**Positive likelihood ratio**	**Post-test probability**
Model 2	5	55.8	9.86	92.6
Model 4	3	55.8	10.25	92.8

**Table 6:** Performance of Approach A (Model 2) in defining the rate of patients unnecessarily explored or inappropriately unexplored

**Model 2**		
**PIV**	**Unnecessarily explored (1−NPV) (%)**	**Inappropriately unexplored (1−PPV) (%)**
0	17.9	28.8
1	25.0	27.6
2	33.3	19.4
3	39.0	18.2
4	48.1	16.3
5	50.0	7.4
6	53.6	5.0
7	54.3	0

**Table 7:** Performances of Approach B (Model 4) in defining the rate of patients unnecessarily explored or inappropriately unexplored

**Model 4**		
**PIV**	**Unnecessarily explored (1−NPV) (%)**	**Inappropriately unexplored (1−PPV) (%)**
0	8.0	37.1
1	25.4	22.6
2	36.3	18.3
3	49.7	7.2
4	54.7	0

**Table 8:** Summary of the studies analysing the performance of computed tomography (CT) scan in the prediction of optimal cytoreduction in ovarian cancer

**Author, year**	**Time**	**Type of study**	**No.**	**% Stage III/IV**	**No. of surgeons**	**Optimal RT (cm)**	**% Optimal debulking**	**CT scan slice thickness**	**CT-based factors**	**CT score**	**Cutoff score**	**NPV**	**PPV**
Nelson *et al* (1993)	December 1985	R	42	81	4	<2	69	NS	8^a^	No	—	95.8	66.7
	May 1991											93.8^b^	66.7^b^
Meyer *et al* (1995)	1989	R	28	57	3	⩽2	57	10 mm	6^c^	Yes^c^	⩾3	76.2	100
	1992											54.5^b^	100^b^
Forstner *et al* (1995)	June 1990 May 1994	P	43	72	NA	⩽2	86	7–10 mm	9^d^	No	—	92.5	100
Bristow *et al* (2000)	July 1997 July 1999	R	41	100	9	⩽1	49	5 mm	13^e^	Yes	⩾4	100	87.5
Byrom *et al* (2002)	January 1998 August 1999	R	77^f^	55	NA	0	36^g^	8–10 mm	2^h^	Yes^i^	5^i^	90	95
Dowdy *et al* (2004)	1996 2001	R	87	100	NA	<1	71	5–10 mm	3^j^	No	—	81^j^	79^j^
Qayyum *et al* (2005)	NA	R	105	77	3	⩽2	80	7–10 mm	15^k^	No	—	96	94
Axtell *et al* (2007)	1999 2005	R	67	100	NA	⩽1	78	5–10 mm	14^l^	No	—	98	46

Abbreviations: NA=not applicable; NS=not specified; P=prospective; R=retrospective.

a CT-assessed criteria for unresectability were as follows: (1) the attachment of omentum to spleen; (2) the presence of >2 cm disease located at any one or more of the following sites: mesentery, gallbladder fossa, pericardiac lymph nodes, pulmonary or pleural nodules, liver surface or parenchyma, suprarenal para-aortic nodes, diaphragm.

b In stage III/IV cases.

c Scores from 0 to 2 were assigned for the presence of disease at the following sites: omentum, liver, para-aortic nodes, diaphragm and lung base, small bowel mesentery, amount of ascites, according to the diameter and extension of the disease.

d The following parameters were used: >2 cm disease located at peritoneum (thickening or implant), at small or larger bowel mesentery, omentum, pelvis (sidewall, parametria, hydroureter), infrarenal or suprarenal nodes, liver (surface or parenchyma), porta hepatis/gallbladder, diaphragm/lung base, inguinal canal, ascites.

e The following parameters were used: >2 cm disease located at mesentery, porta hepatis, lesser sac, intersegmental fissure, dome of the liver, gastrosplenic ligament, diaphragm, nodes at and above the celiac axis, presacral extraperitoneal disease.

f Including also benign (*n*=26), and early-stage cancer (*n*=23).

g 36% in the whole series (52 out of 77), and 10.7% in stage III–IV cancer (3 out of 28).

h Only CT-assessed omental cake and presence of mesenteric disease were independent predictors of unresectability;

i The score also included age and Ca125 levels.

j Multivariate analysis identified three variables (diffuse peritoneal carcinomatosis, ascites, and diaphragm involvement) as the only significant predictors of unresectability; NPV and PPV refer to the model including these three parameters.

k CT-assessed criteria for unresectability were as follows: presence of >2 cm disease located at any one or more of 14 critical sites: porta hepatis, intersegmental fissure, gallbladder fossa, subphrenic space, gastrohepatic and gastrosplenic ligaments, lesser sac, small bowel mesentery, dome of the liver surface, suprarenal para-aortic nodes, celiac axis supradiaphragmatic involvement, liver, abdominal wall invasion.

l Multivariate analysis including 14 CT-based variables and four clinical parameters identified only two variables (tumour>2 cm at the large bowel mesentery and diaphragm) as the only significant predictors of unresectability; NPV and PPV refer to the model including these two parameters.

## References

[bib1] Aletti GD, Dowdy JL, Podratz KL, Cliby WA (2007) Relationship among surgical complexity, short-term morbidity and overall survival in primary surgery for advanced ovarian cancer. Am J Obstet Gynecol 197: 676.e1–676.e7 18060979 10.1016/j.ajog.2007.10.495

[bib2] Axtell AE, Lee MH, Bristow RE, Dowdy SC, Cliby WA, Raman S, Weaver JP, Gabbay M, Ngo M, Lentz S, Cass I, Li AJ, Karlan BY, Holschneider CH (2007) Multi-institutional reciprocal validation study of computed tomography predictors of suboptimal primary cytoreduction in patients with advanced ovarian cancer. J Clin Oncol 25: 384–389 17264334 10.1200/JCO.2006.07.7800

[bib3] Bristow RE, Duska LR, Lambrou NC, Fishman EK (2000) A model for predicting surgical outcome in patients with advanced ovarian carcinoma using computed tomography. Cancer 89: 1532–1540 11013368 10.1002/1097-0142(20001001)89:7<1532::aid-cncr17>3.0.co;2-a

[bib4] Bristow RE, Tomacruz RS, Armstrong DK, Trimble EL, Montz FJ (2002) Survival effect of maximal cytoreductive surgery for advanced ovarian carcinoma during the platinum era: a meta-analysis. J Clin Oncol 20: 1248–1259 11870167 10.1200/JCO.2002.20.5.1248

[bib5] Brun JL, Rouzier R, Uzan S, Daraï E (2008) External validation of a laparoscopic-based score to evaluate resectability of advanced ovarian cancers: clues for a simplified score. Gynecol Oncol 110: 354–359 18572226 10.1016/j.ygyno.2008.04.042

[bib6] Byrom J, Widjaja E, Redman CWE, Jones PW, Tebby S (2002) Can preoperative computer tomography predict resectabiity of ovarian carcinoma at primary laparotomy? Br J Obstet Gynecol 109: 369–375 10.1111/j.1471-0528.2002.01216.x 12013156

[bib7] Chi DS, Venkatraman ES, Masson V, Hoskins WJ (2000) The ability of preoperative serum CA-125 to predict optimal primary tumor cytoreduction in stage III epithelial ovarian carcinoma. Gynecol Oncol 77: 227–231 10785469 10.1006/gyno.2000.5749

[bib8] Chi DS, Zivanovic O, Palayekar MJ, Eisenhauer EL, Abu-Rustum NR, Sonoda Y, Levine DA, Leitao MM, Brown CL, Barakat RR (2009) A contemporary analysis of the ability of preoperative serum CA-125 to predict primary cytoreductive outcome in patients with advanced ovarian, tubal and peritoneal carcinoma. Gynecol Oncol 112: 6–10 19100916 10.1016/j.ygyno.2008.10.010

[bib9] Cooper BC, Sood AK, Davis CS, Ritchie JM, Sorosky JL, Anderson B, Buller RE (2002) Preoperative CA-125 levels: an independent prognostic factor for epithelial ovarian cancer. Obstet Gynecol 100: 59–64 12100804 10.1016/s0029-7844(02)02057-4

[bib10] Cox DR (1970) Analysis of Binary Data. Methven: London

[bib11] Deeks JJ, Altman DG (2004) Diagnostic tests 4: likelihood ratios. Br Med J 329: 168–169 15258077 10.1136/bmj.329.7458.168 PMC478236

[bib12] Dowdy SC, Mullany SA, Brandt KR, Huppert B, Cliby WA (2004) The utility of computed tomography scans in predicting suboptimal cytoreductive surgery in women with advanced ovarian carcinoma. Cancer 101: 346–352 15241833 10.1002/cncr.20376

[bib13] Eisenkop SM, Spirtos NM (2001) What are the current surgical objectives, strategies, and technical capabilities of gynecologic oncologists treating advanced epithelial ovarian cancer? Gynecol Oncol 82: 489–497 11520145 10.1006/gyno.2001.6312

[bib14] Eisenkop SM, Spirtos NM, Lin WCM (2006) ‘Optimal’ cytoreduction for advanced epithelial ovarian cancer: a commentary. Gynecol Oncol 103: 329–335 16876853 10.1016/j.ygyno.2006.07.004

[bib15] Fagotti A, Ferrandina G, Fanfani F, Garganese G, Vizzielli G, Carone V, Salerno MG, Scambia G (2008) Prospective validation of a laparoscopic predictive model for optimal cytoreduction in advanced ovarian carcinoma. Am J Obstet Gynecol 199: 642.e1–642.e6 18801470 10.1016/j.ajog.2008.06.052

[bib16] Fagotti A, Ferrandina MG, Fanfani F, Ercoli A, Lorusso D, Rossi M, Scambia G (2006) A laparoscopy-based score to predict surgical outcome in patients with advanced ovarian carcinoma: a pilot study. Ann Surg Oncol 13: 1156–1161 16791447 10.1245/ASO.2006.08.021

[bib17] Fanfani F, Ferrandina G, Corrado G, Fagotti A, Zakut HV, Mancuso S, Scambia G (2003) Impact of interval debulking surgery on clinical outcome in primary unresectable FIGO Stage IIIC ovarian cancer patients. Oncology 65: 16–22 10.1159/000074644 14707451

[bib18] Forstner R, Hricak H, Occhipinti KA, Powell CB, Frankel SD, Stern JL (1995) Ovarian cancer: staging with CT and MR imaging. Radiology 197: 619–626 7480729 10.1148/radiology.197.3.7480729

[bib19] Gemer O, Gdalevich M, Ravid M, Piura B, Rabnovich A, Gasper T, Khashper A, Voldarsky M, Linov L, Ben Shacar I, Anteby EY, Lavie O (2009) A multicenter validation of computerized tomography models as predictors of non-optimal primary cytoreduction of advanced epithelial ovarian cancer. Eur J Surgical Oncol 1–4 10.1016/j.ejso.2009.03.002 19329270

[bib20] Hanley JA, McNeil BJ (1982) The meaning and use of the area under a receiver operating characteristics (ROC) curve. Radiology 143: 29–36 7063747 10.1148/radiology.143.1.7063747

[bib21] Hunter RW, Alexander ND, Soutter WP (1992) Meta-analysis of surgery in advanced ovarian carcinoma: is maximum cytoreductive surgery an independent determinant of prognosis? Am J Obstet Gynecol 166: 504–511 1531572 10.1016/0002-9378(92)91658-w

[bib22] Memarzadeh S, Lee SB, Berek JS, Farias-Esner R (2003) CA-125 levels are a weak predictor of optimal cytoreductive surgery in patients with advanced epithelial ovarian cancer. Int J Gynecol Cancer 13: 120–124 12657110 10.1046/j.1525-1438.2003.13019.x

[bib23] Meyer JI, Kennedy AW, Friedman R, Hricak H, Occhipinti KA, Bethan Powell C, Frankel SD, Stern JL (1995) Ovarian carcinoma: value of CT in predicting success of debulking surgery. Am J Roentgenol 165: 875–878 7676985 10.2214/ajr.165.4.7676985

[bib24] Nelson BE, Rosenfield AT, Schwartz PE (1993) Preoperative abdominopelvic computed tomographic prediction of optimal cytoreduction in epithelial ovarian carcinoma. J Clin Oncol 11: 166–172 8418230 10.1200/JCO.1993.11.1.166

[bib25] Nickles Fader A, Rose P (2007) Role of surgery in ovarian carcinoma. J Clin Oncol 25: 2873–2883 17617518 10.1200/JCO.2007.11.0932

[bib26] Qayyum A, Coakley FV, Westphalan AC, Hricak H, Okuno WT, Powell B (2005) Role of CT and MRI imaging in predicting optimal cytoreduction of newly diagnosed primary epithelial ovarian cancer. Gynecol Oncol 96: 301–306 15661212 10.1016/j.ygyno.2004.06.054

[bib27] Risum S, Høgdall C, Loft A, Berthelsen AK, Høgdall E, Nedergaard L, Lundvall L, Engelholm SA (2008) Prediction of suboptimal primary cytoreduction in primary ovarian cancer with combined positron emission tomography/computed tomography-a prospective study. Gynecol Oncol 8: 265–270 10.1016/j.ygyno.2007.11.002 18055006

[bib28] Saygili U, Guclu S, Uslu T, Erten O, Dogan E (2002) Can serum CA-125 levels predict the optimal primary cytoreduction in patients with advanced ovarian carcinoma? Gynecol Oncol 86: 57–61 12079301 10.1006/gyno.2002.6719

[bib29] Vergote I, Trope’ CG, Amant F, Kristensen GB, Sardi JE, Ehelen T, Jonhson N, Verheijen RHM, Van der Burg MEL, Lacave AJ, Benedetti Panici P, Kenter GG, Casado A, Mendiola C, Coens C, Stuart G, Pecorelli S, Reed NS (2008) EORTC-GCG/NCIC-CTG randomized trial comparing primary debulking surgery with neoadjuvant chemotherapy in stage IIIC-IV ovarian, fallopian tube and peritoneal cancer. Biennial International Gynecological Cancer Society Meeting; Bangkok, 25–28 October

[bib30] Vergote I, van Gorp T, Amant F, Neven P, Barteloot P (2005) Neoadjuvant chemotherapy for ovarian cancer. Oncology 19: 1615–1622 16396153

[bib31] Wakabayashi MT, Lin PS, Hakim AA (2008) The role of cytoreductive/debulking surgery in ovarian cancer. J Natl Compr Cancer Network 6: 803–810 10.6004/jnccn.2008.0060 18926091

